# Impact on health‐related quality of life and symptoms of anxiety and depression after 32 weeks of Dupilumab treatment for moderate‐to‐severe atopic dermatitis

**DOI:** 10.1111/dth.15407

**Published:** 2022-03-03

**Authors:** Marco Miniotti, Giulia Lazzarin, Michela Ortoncelli, Luca Mastorino, Simone Ribero, Paolo Leombruni

**Affiliations:** ^1^ ‘Rita Levi Montalcini’ Department of Neuroscience University of Turin Turin Italy; ^2^ Medical Science Department, Dermatology Clinic University of Turin Turin Italy

**Keywords:** anxiety, atopic dermatitis, depression, Dupilumab, patient‐reported outcomes, quality of life

## Abstract

Dupilumab is the first biological agent approved for treatment of moderate‐to‐severe atopic dermatitis (AD). Evidence of Dupilumab effectiveness on psychological outcomes beyond 16 weeks of treatment from real‐life settings is lacking. To evaluate the effectiveness of Dupilumab treatment up to 32 weeks, focusing health‐related quality of life and psychological outcome of patients with moderate‐to‐severe AD. An observational prospective cohort study was conducted in a real‐life setting at an Italian tertiary centre. Assessment of outcome measures was carried out at baseline, after 16 and 32 weeks of treatment. A total of 171 patients were included. EASI‐75 and EASI‐90 were achieved in 85% and 60% of the participants, respectively, after 16 weeks, and in 89.6% and 69.8% after 32 weeks of treatment. Significant improvements (*p* < 0.001; *r* = 0.57–0.95) were found after 16 weeks for each outcome considered, including clinician and patient‐reported measures of AD severity and scales of health‐related quality of life and psychological morbidity, and maintained up to 32 weeks. Further analysis revealed that patients' quality of life was more associated with the subjective perception of disease severity rather than objective measures and suggested a possible different response to treatment based on the age of AD onset. Dupilumab was confirmed to be rapid, effective and safe in patients with moderate‐to‐severe AD. Its positive impact on psychological outcomes up to 32 weeks was ascertained here, adding new evidence on the need to consider subjective factors affecting patients' perception of disease severity in evaluating the response to treatment.

## 1 INTRODUCTION

Atopic dermatitis (AD) is a common and chronic immune‐mediated inflammatory skin disease characterized by erythematous and eczematous lesions with intense pruritus, affecting 7–10% of adults.^1^ AD is associated with debilitating effects on health‐related quality of life including pain, sleep disturbances, and psychological morbidity.^2^, ^3^ Systemic treatment for moderate‐to‐severe AD was limited because of the risk/ benefit ratio associated, and a need for safe and effective solutions for those patients who did not respond to topic medications or immunosuppressants was present.^4^, ^5^

A significant change has occurred when a fully human monoclonal antibody Dupilumab was approved by US Food and Drug Administration (FDA) and the European Medicines Agency (EMA) for the treatment of patients with moderate‐to‐severe AD nonresponsive to cyclosporine or other treatments.^6^ Dupilumab targets the interleukin (IL‐4) receptor‐alpha (IL‐4Rα) subunit and inhibits signaling of cytokines IL‐4 and IL‐13, both driving at least Type 2 inflammation including AD.^7^ The effectiveness and safety of Dupilumab were evaluated in placebo‐controlled Phase 3 clinical trials in adults with moderate‐to‐severe AD inadequately controlled with topical treatment or intolerant to immunosuppressants or corticosteroids.^8^, ^9^, ^10^, ^11^ Results from these clinical trials were robust and consistent in showing that, compared with a placebo, Dupilumab significantly improves objective signs of AD, symptoms related including pruritus, pain, sleep disturbance, anxiety and depression, and multiple areas of patients' health‐related quality of life.

Despite this evidence, findings resulting from real‐life experience reflecting actual conditions encountered in daily practice are limited to date, especially beyond 16 weeks of treatment. This study was precisely conducted to obtain further insight into the effectiveness of long‐term Dupilumab treatment and its impact on health‐related quality of life and psychological outcome of moderate‐to‐severe AD patients.

## 2 METHODS

### 2.1 Participants and procedures

Prospectively collected data from a tertiary referral centre for AD in northern Italy (Dermatology Clinic of the University of Turin) was analyzed in this study. Consecutive patients more than 18 years old with a clinical or histological diagnosis of AD were enrolled between January and September 2019. Patients received Dupilumab for moderate‐to‐severe AD (EASI ≥ 24 or less in cases with eczema in sensitive areas) due to cyclosporine inefficiency, loss of efficiency, or contraindication.

Assessments of outcome measures were carried out at baseline (T0), after 14 weeks (T1), and after 32 weeks (T2) of Dupilumab treatment.

This study was approved by the local ethical committee of the Turin University Hospital (No. CS2/1359).

### 2.2 Measures

Extent and severity of AD were evaluated by dermatologists through the Eczema Area and Severity Index (EASI)^12^ and the SCORing atopic dermatitis (SCORAD Index).^13^ EASI score ranged from 0 to 72 and was categorized as follows: 0 = clear, 0.1–1.0 = almost clear, 1.1–7.0 = mild, 7.1–21.0 = moderate, 21.1–50 = severe, 50.1–71 = very severe.^14^ Improvements of 75% (EASI‐75) and 90% (EASI‐90) in EASI score from baseline were considered as parameters for minimal clinically important difference (MCID).^15^ SCORAD score ranged from 0 to 103, with higher scores indicating more severe AD. A reduction of 8.2 points from baseline score was considered as MCID for SCORAD index.^15^

Patients self‐reported AD symptoms through the Patient‐Reported Eczema Measure (POEM)^16^ (scores ranged from 0 to 28) and Numerical Rating Scales (0–10) for pruritus (Itch‐NRS) and sleep disorders (Sleep‐NRS). A reduction of 3.4 points from baseline score was considered as MCID for POEM.^15^ AD impact on health‐related quality of life was self‐evaluated by patients through the Dermatology Quality of Life Index (DLQI),^17^ and the Short Form Health Survey (SF‐36).^18^ DLQI scores ranged from 0 to 30 and was categorized as follows: 0–1 = no effect, 2–5 = small effect; 6–10 = moderate effect; 11–20 = large effect; 21–30 = extremely large effect.^19^ SF‐36 scores rangd from 0 to 100 for each of the eight subscales (Physical functioning, Role functioning, Bodily pain, General health, Vitality, Social functioning, Emotional functioning, Mental health) and the two summary measures (Physical Health, Mental Health). Symptoms of anxiety and depression were assessed through the Hospital Anxiety and Depression Scale (HADS).^20^ HADS scores ranged from 0 to 21 for both the subscales (Anxiety, Depression), with a score of 11 or above indicating clinical symptoms.

### 2.3 Statistical analyses

Data were analyzed with SPSS software v. 25.0 for Mac (IBM, Armonk, New York). Two‐sided *p*‐value < 0.05 was considered statistically significant.General descriptive statistics were calculated to synthetize data exposition; median and interquartile ranges were used for non‐normally distributed variables. One‐way repeated measure analyses of variance (ANOVA) with the Bonferroni post hoc test to take account of pairwise comparisons were run to estimate changes in outcomes variables throughout assessment points (i.e., baseline, after 4 months, and after 8 months). The Friedman test was used as nonparametric alternative and Wilcoxon signed‐rank tests were run for pairwise comparisons. Effect‐size estimate *r* was calculated to standardize the size of the effects observed. Multiple linear regressions were run to study the effect of outcome variables on a DLQI score at baseline and after 16 weeks; semi‐partial correlation coefficients were calculated to estimate the amount of the variance in DLQI accounted by each independent outcome variable individually. Two‐way and three‐way mixed ANOVAs were performed to determine differences between independent groups (formed by demographic characteristics, clinical features, and combinations of these) in changes of outcome variables over time (baseline>T1). Follow‐up tests were performed to explore main effects.

## 3 RESULTS

### 3.1 Descriptive analysis

The sample considered included 171 participants, 91 (53.2%) males and 80 (46.8%) females, with a mean age of 39.3 ± 16.2. Detailed sample characteristics are reported in Table 1. No significant between‐group differences concerning demographic or dermatological and clinical features existed at baseline on the outcome variables.

**TABLE 1:** Baseline characteristics of the whole sample

Participants characteristics	*N* = 171
Age, *m*(*SD*)	39.3 ± 16.2
Sex, *n* (%)	
M	91(53.2)
F	80(46.8)
Education, *n* (%)	
Primary/ secondary school	25(14.6)
High school	98(57.3)
University degree	48(28.1)
Age of AD onset, *n* (%)	
Within the first year of age	98(57.3)
≤ 18	26(15.2)
19–30	18(10.5)
≥ 31	29(17.0)
Localization of AD, *n* (%)	
Head and neck	5(2.9)
Upper limbs	3(1.8)
Lower limbs	2(1.2)
More than half of the body	71(41.5)
Diffuse	90(52.6)
Previous treatment, *n* (%)	
Topical steroids	167(98.8)
Topical calcineurin inhibitors	80(47.3)
Systemic steroids	163(96.4)
Cyclosporine	152(89.9)
Phototherapy	22(13.0)
Phototype, *n* (%)	
0	2(1.2)
I	6(3.5)
II	72(42.1)
III	84(49.1)
IV	7(4.1)
Hair color, *n* (%)	
White	5(2.9)
Brown	141(82.5)
Black	11(6.4)
Blonde	12(7.0)
Red	2(1.2)
Eyes color, *n* (%)	
Brown	121(70.8)
Blue	31(18.1)
Green	19(11.1)
Allergies, *n* (%)	46(27.2)
Asthma, *n* (%)	42(24.9)
Rhinoconjunctivitis, *n* (%)	11(6.5)
Alopecia, *n* (%)	5(3.0)
Psychiatric disorder, *n* (%)	8(4.7)
Smoking status, *n* (%)	
Non‐/Ex‐smoker	118(69.0)
< 10 cigarettes/die	40(23.4)
≥ 10 cigarettes/die	13(7.6)
Alcohol consumption, *n* (%)	
None	73(42.7)
Occasionally	86(50.3)
Habitually	12(7.0)
BMI, *m*(SD)	23.4(4.2)

EASI‐75 and EASI‐90 were achieved in 85% and in 60% of the participants, respectively, after 16 weeks, which increased to 89.6% and 69.8% after 32 weeks of Dupilumab treatment. MCID for the SCORAD index was reached by 98.2% of the participants after 16 weeks and by the total sample after 32 weeks of treatment. For POEM, MCID was achieved by 89.3% of the participants after 16 weeks of treatment and 90.2% of them after 32 weeks. Similarly, MCID for DLQI was achieved by 84.4% of the participants after 16 weeks and by 87.5% after 32 weeks of treatment.

### 3.2 Longitudinal analysis

Results of one‐way repeated measures ANOVAs and Friedman tests are reported in Table 2, together with descriptive statistics for the outcome variables at baseline, after 16 and 32 weeks of Dupilumab treatment. Statistically significant improvements (*p* < 0.001) were found for each outcome variable considered (i.e. EASI, SCORAD, POEM, DLQI, Itch‐NRS, Sleep‐NRS, HADS, and SF‐36). Post hoc analyses revealed a constant pattern of change over time that provided significant improvements in outcomes concentrated in the first 16 weeks and a consequent stabilization of them up to 32 weeks of treatment. Effect sizes for the improvements from baseline to 16 weeks were large, as they ranged from 0.57 to 0.95.

**TABLE 2:** Effectiveness outcomes of Dupilumab after 16 weeks (T1) and after 32 weeks (T2): one‐way repeated measures ANOVA with post hoc tests and Friedman's test with Wilcoxon signed‐rank tests as post hoc tests

Outcome variable	Baseline T0	16 weeks T1	32 weeks T2	GLM Rep Meas/ Friedman test^a^	Post hoc test/ Wilcoxon signed‐rank test^b^	
					T0/T1	T1/T2
	*m* ± *SD*/M(IQR)	*m* ± *SD*/M(IQR)	*m* ± *SD*/M(IQR)	*p*‐Value	*p‐*Value, *r*	*p‐*Value
EASI	26.7 ± 11.3	2.9 ± 3.7	2.6 ± 2.9	<0.001	<0.001, 0.91	0.461
SCORAD	62.2 ± 11.8	17.1 ± 11.8	14.8 ± 11.5	<0.001	<0.001, 0.95	0.080
POEM	22.0(16.0–25.0)	5.0(2.0–11.0)	5.0(2.0–11.0)	<0.001	<0.001, 0.88	1.000
DLQI	15.9 ± 7.0	4.1 ± 4.4	3.9 ± 4.7	<0.001	<0.001, 0.86	0.802
Itch‐NRS	10.0(8.0–10.0)	2.0(1.0–4.0)	3.0(1.0–4.0)	<0.001	<0.001, 0.92	0.483
Sleep‐NRS	8.0(5.0–10.0)	0.0(0.0–1.7)	0.0(0.0–0.0)	<0.001	<0.001, 0.87	1.000
HADS‐A	7.0(5.0–11.0)	5.0(3.0–7.0)	4.0(2.0–7.0)	<0.001	<0.001, 0.62	0.111
HADS‐D	6.0(3.0–9.0)	3.0(2.0–6.0)	3.0(1.0–6.0)	<0.001	<0.001, 0.57	1.000
SF‐36‐PF	85.0(70.0–95.0)	95.0(90.0–100.0)	100.0(90.0–100.0)	<0.001	<0.001, 0.57	0.459
SF‐36‐RP	50.0(0.0–75.0)	100.0(75.0–100.0)	100.0(75.0–100.0)	<0.001	<0.001, 0.62	0.950
SF‐36‐BP	45.5(24.4–67.8)	93.3(74.4–100.0)	93.3(82.2–100.0)	<0.001	<0.001, 0.79	0.819
SF‐36‐GH	48.2 ± 21.8	64.6 ± 20.2	65.4 ± 18.7	<0.001	<0.001, 0.62	0.591
SF‐36‐V	47.7 ± 17.4	63.6 ± 15.5	67.3 ± 15.5	<0.001	<0.001, 0.62	0.112
SF‐36‐SF	50.0(37.5–75.0)	87.5(62.5–100.0)	87.5(75.0–100.0)	<0.001	<0.001, 0.73	0.787
SF‐36‐RE	33.3(0.0–100.0)	100.0(100.0–100.0)	100.0(100.0–100.0)	<0.001	<0.001, 0.66	1.000
SF‐36‐MH	57.0 ± 20.2	73.1 ± 14.5	73.4 ± 16.4	<0.001	<0.001, 0.62	1.000
Adv. event	*n* (%)	*n* (%)	*n* (%)	Bring to discontinuation, *n* (%)		
IS reaction	/	2(1.2)	/			
Herpes	/	6(3.6)	/			
Blef./Conj.	/	18(10.7)	19(11.2)		4(2.3)	
Cephalea	/	5(3.0)	/			
Itchy eye	/	11(6.5)	/			
To report						
Ineffectiveness					3(1.7)	
Pregnancy					3(1.7)	
Bladder cancer					1(0.6)	
Arthromyalgia					1(0.6)	
Skin rashes					1(0.6)	

Abbreviations: Adv. event, adverse event; Blef./Conj., blepharitis or conjunctivitis; DLQI, Dermatology Life Quality Index; EASI, Eczema Area and Severity Index; HADS‐A, Hospital Anxiety and Depression Scale—Anxiety subscale; HADS‐D, Hospital Anxiety and Depression Scale—Depression subscale; IS reaction, Reaction to the injection site; Itch‐NRS, Numerical Rating Scale for itch; *m* ± *SD*/M(IQR), mean ± standard deviation/median (interquartile range); POEM, Patient Oriented Eczema Measure; *r*, effect size; SCORAD, SCOring Atopic Dermatitis index; SF‐36‐BP, Short Form Health Survey—bodily pain subscale; SF‐36‐GH, Short Form Health Survey—general health subscale; SF‐36‐MH, Short Form Health Survey—mental health subscale; SF‐36‐PF, Short Form Health Survey—physical functioning subscale; SF‐36‐RE, Short Form Health Survey—role emotional subscale; SF‐36‐RP, Short Form Health Survey—role physical subscale; SF‐36‐SF, Short Form Health Survey—social functioning subscale; SF‐36‐V, Short Form Health Survey—vitality subscale; Sleep‐NRS, Numerical Rating Scale for sleep disorders.

^a^ *P* value of one‐way repeated measure ANOVA model or Friedman's test.

^b^ Post hoc test for one‐way repeated measure ANOVA or Wilcoxon signed‐rank test as post hoc test for Friedman's test (Bonferroni correction with alpha set at 0.017).

AEs reported were concentred from baseline to 16 weeks of treatment, except from blepharitis and/or conjunctivitis that persisted until 32 weeks for 11% of the participants. Outcomes did not differ between patients with or without blepharitis and/or conjunctivitis; nevertheless, four patients with eye disorders discontinued the therapy. AEs are detailed in Table 2.

### 3.3 Regression analysis

The model performed with outcome scores at baseline was statistically significant (*F* = 13.139, *p* < 0.001) and explained 49% of the total variance in DLQI scores with three significant predictors: POEM (*p* = 0.001), Sleep‐NRS (*p* = 0.031) and SF‐36 Physical Health summary measure (*p* = 0.001). Adjusting for other independent variables, POEM, Sleep‐NRS and SF‐36 Physical Health singly accounted for 4.7%, 2.0% and 4.6% of the variance in DLQI, respectively. The model performed with outcome scores after 16 weeks of treatment was statistically significant (*F* = 17.028, *p* < 0.001) and explained 62% of the total variance in DLQI scores with three significant predictors: POEM (*p* = 0.015), Itch‐NRS (*p* = 0.001) and HADS‐A (*p* = 0.019). Adjusting for other independent variables, POEM, Itch‐NRS and HADS‐A singly accounted for 2.5%, 5.2%, and 2.3% of the variance in DLQI, respectively. A more detailed summary of regression analysis is presented in Table 3.

**TABLE 3:** Effect of outcome measures on DLQI scores: multiple linear regressions

Outcome variable	Baseline (T0)		16 weeks (T1)	
	*B*	sr	*B*	sr
Constant	15.256*		9.647*	
EASI	−0.031	−0.032	0.044	0.021
SCORAD	0.044	0.051	−0.006	−0.008
POEM	0.307*	−0.063	0.197*	0.158
Itch‐NRS	−0.361	0.141	0.744*	0.228
Sleep‐NRS	0.466*	0.218	0.228	0.071
HADS‐A	0.146	0.057	0.295*	0.152
HADS‐D	−0.169	−0.067	0.019	0.009
SF‐36 Physical Health	−0.126*	−0.214	−0.006	−0.013
SF‐36 Mental Health	−0.029	−0.049	−0.006	−0.013

Abbreviations: *B*, unstandardized regression coefficient; EASI, Eczema Area and Severity Index; HADS‐A, Hospital Anxiety and Depression Scale—Anxiety subscale; HADS‐D, Hospital Anxiety and Depression Scale—depression subscale; Itch‐NRS, Numerical Rating Scale for itch; POEM, Patient Oriented Eczema Measure; SCORAD, SCOring Atopic Dermatitis index; SF‐36 Mental Health, Short Form Health Survey Mental Health summary; SF‐36 Physical Health, Short Form Health Survey Physical Health summary; Sleep‐NRS, Numerical Rating Scale for sleep disorders; sr, semipartial regression coefficient.

* Significant at 0.05 level.

### 3.4 Mixed‐design analysis

Two‐way mixed ANOVA revealed a significant two‐way interaction between time (baseline > T1) and age of AD onset on SF36 Physical Health summary measure (*F* = 3.407, *p* = 0.020, partial η^2^ = 0.091) controlling for EASI score. There was a significant difference in physical health between different ages of AD onset after 16 weeks of treatment (*F* = 3.169, *p* = 0.027, partial *η* ^2^ = 0.081). Physical health was significantly lower in the ≥31 age group compared to the other categories of age of AD onset (*p* = 0.025).

Three‐way mixed ANOVA revealed significant three‐way interactions among time (baseline > T1), sex, and age of AD onset on SF36 Mental Health summary measure (*F* = 2.729, *p* = 0.044, partial *η* ^2^ = 0.077) controlling for EASI score. There was a significant two‐way interaction between sex and age of AD onset after 16 weeks of treatment (*F* = 3.553, *p* = 0.017, partial *η* ^2^ = 0.093). Significant simple main effect of age of AD onset at T1 was observed for females (*F* = 3.724, *p* = 0.014, partial *η* ^2^ = 0.097). Mental health was lower in females with age of AD onset ≥31 compared to those with age of AD onset < 18 (*p* = 0.015) and within the first year of age (*p* = 0.027).

## 4 DISCUSSION

This study showed that Dupilumab treatment of moderate‐to‐severe AD patients has resulted in rapid improvement of objective signs of the disease, as ascertained by the sharp and fast drop of EASI and SCORAD indexes, and of key outcomes for such patients including self‐perception of eczema severity, pruritus, sleep disturbance, symptoms of anxiety and depression, and multiple aspects of health‐related quality of life. The extent of the improvement in the outcomes after 16 weeks of treatment from baseline was proven by the large effect sizes found and was maintained at 32 weeks of treatment.

The results presented in this study are noteworthy as they not only confirm findings from previous real‐life studies as regards the clear and rapid effectiveness of Dupilumab,^21^, ^22^, ^23^, ^24^, ^25^, ^26^, ^27^, ^28^ but also produced expected data about the consolidation over time of the multiple improvements in both clinician‐ and patient‐reported measures showed after 16 weeks in previous studies^21^, ^22^, ^23^, ^24^, ^25^, ^26^, ^27^, ^28^ since, to date, very little data is available about the effectiveness of this drug beyond 4 months.^29^, ^30^, ^31^ Moreover, MCID percentages reported in this study are slightly higher than those documented in previous research. In this study, EASI‐75 was achieved by 85% of the participants after 16 weeks and approximately by 90% after 32 weeks of treatment, similarly to another Italian study in which 88% of patients achieved EASI‐75 after 4 months.^28^ Differently, in clinical trials SOLO 1,^5^ SOLO 2,^5^ CHRONOS^9^ and CAFÉ,^10^ EASI‐75 was achieved by 51%, 44%, 69%, and 61.6%, respectively. In real‐life studies, EASI‐75 was attained after 3 months by 48.8% of the participants in a multicentre retrospective French study^25^ and by 63.3% in a small‐size Danish cohort^24^; after 4 months, EASI‐75 was attained by 72.7% in a single‐center Italian study,^21^ by 60.6%^23^ and 81.5%^22^ in two different multicentre retrospective Italian cohorts, and63.6% in a single‐centre retrospective study in Korea.^27^

Only three participants (1.7%) in this study did not report any improvement in AD signs and symptoms, less than reported in previous multicentre real‐life studies.^25^, ^29^ Single‐centre studies like this typically ensure greater treatment uniformity and adherence and this should be kept in mind when weighing up the result.

In line with the literature, we reported that blepharitis with or without mild conjunctivitis was the most frequent AE related to Dupilumab treatment, being reported indeed by 19 participants, with only 4 of whom discontinued the therapy due to this AE. These data are lower than that reported in other real‐life studies,^24^, ^25^, ^26^ higher than reported in a study conducted in Korea,^27^ and very similar to previous Italian studies.^21^, ^22^, ^23^, ^28^ Dupilumab‐induced conjunctivitis seemed to be specific to AD, since it was not reported in clinical trials with other populations (asthma, nasal polyposis) treated with this monoclonal antibody.^32^, ^33^ In spite of the occurrence of conjunctivitis in patients receiving Dupilumab having been linked with recurring atopic conjunctivitis,^22^, ^25^ and other atopic comorbidities,^34^ pathophysiology of eye disorders related to treatment with this drug remained unclear. Future studies should address etiology and therapy of this adverse phenomenon since, since, although it does not significantly affect the quality of life of most patients, in a few cases it could lead to treatment discontinuation as reported in this study.

Output from regression analyses showed the singular contribution of each outcome variable in determining DLQI scores and revealed that the measures of subjective perception of symptom severity (i.e. POEM, NRS for pruritus and sleep disturbance and SF‐36 Physical Health summary measure) were the most significant predictors of the impairment in quality of life experienced by patients with moderate‐to‐severe AD, independently of objective measures of disease severity, such as EASI and SCORAD indexes. These results corroborated previous observations^27^ and suggest that, first, physical signs of skin lesions and subjective experience of symptoms might be not related to each other and, second, the latter has a major role in determining the quality of life the patients perceive. In turn, this finding suggests that annoying symptoms of the disease should not be neglected or overlooked even in the face of low EASI and SCORAD scores, confirming the need to consider patient‐reported outcome measures (PROMs) to understand fully the patient's overall experience of the disease. The recent findings about discrepancy in PROMs betterments among culturally different AD populations treated with Dupilumab, in face of comparable improvements in EASI scores,^35^ are in line with what is stated here.

The study of between‐group differences across changes in outcome variables from baseline to 16 weeks of treatment suggested that, controlling for changes in EASI scores, the improvement in physical health was lower in patients with AD onset in adulthood (i.e., ≥31 years old) and that the improvement in mental health was lower in females with AD onset in adulthood. The nonsignificant interaction of EASI scores seemed to exclude that these sub‐populations were Dupilumab nonresponders. The absence of comparable data in the literature does not allow to argue further these observations. Future investigations will eventually confirm these findings and provide possible explanations.

The main strengths of this real‐life study are that the participants were not selected and represented a large sample of the total Italian AD population treated with Dupilumab, to date. Moreover, this study added expected evidences about consolidation beyond 4 months of well‐documented rapid improvements in clinician‐ and patient‐reported measures in patients with moderate‐to‐severe AD treated with this drug. The main limitation of this study is the absence of control group and/or comparisons with other systemic therapies.

## 5 CONCLUSION

This study has shown that Dupilumab treatment in a routine clinical setting can lead to rapid and significant improvement in signs and symptoms of moderate‐to‐severe AD, confirming previous findings from clinical trials and real‐life studies, and that this improvement is sustained up to 32 weeks for psychological outcomes, adding new evidences about long‐term effectiveness of this drug. In line with previous research, Dupilumab demonstrated favorable safety and tolerability profile in this study. However, eye complaints were confirmed as the most frequent AE reported, even if it did not lead to treatment discontinuation in the vast majority of the cases. Findings about links among psychological outcomes add new evidence on the need to consider subjective factors affecting patients’ perception of the disease severity, and in evaluating its severity and their response to treatment.

## CONFLICT OF INTEREST

The authors declare that they have no conflict of interest, financial or others.

## AUTHOR CONTRIBUTIONS

Marco Miniotti designed and conducted the study, analyzed and interpreted the data and wrote the manuscript. Giulia Lazzarin collected the data. Michela Ortoncelli critically reviewed the manuscript. Luca Mastorino critically reviewed the manuscript. Simone Ribero supervised the study and critically reviewed the manuscript. Paolo Leombruni supervised the study and critically reviewed the manuscript. All the authors approved the final version of the manuscript.

## Data Availability

The data that support the findings of this study are available from the corresponding author upon reasonable request.
